# Supplementary figures and images for: Elastic Shape Analysis of Surfaces with Second-Order Sobolev Metrics: A Comprehensive Numerical Framework
Source: Int J Comput Vis. 2023 Jan 21;131(5):1183–209. doi: 10.1007/s11263-022-01743-0 (PMC10102155; doi:10.1007/s11263-022-01743-0)

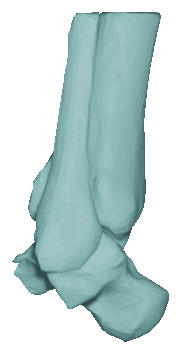

Supplement: Supplementary file 1 — (zip 13912 KB) [file 11263_2022_1743_MOESM1_ESM.zip › 11263_2022_1743_MOESM1_ESM/ankle.gif]

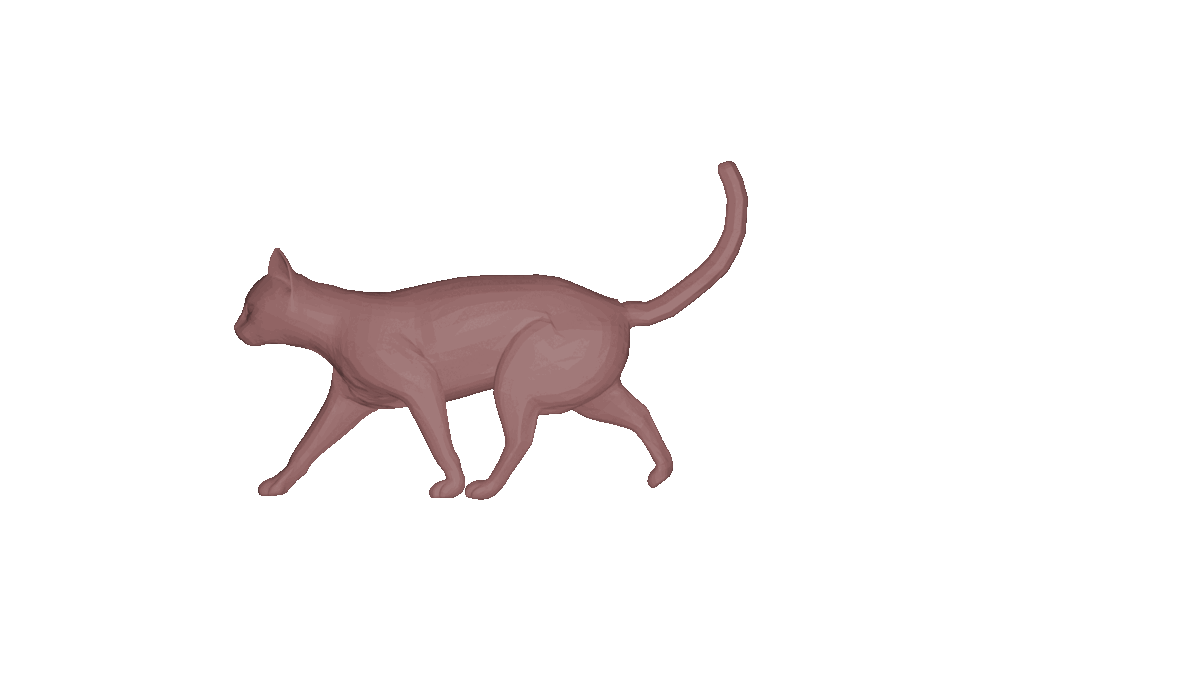

Supplement: Supplementary file 1 — (zip 13912 KB) [file 11263_2022_1743_MOESM1_ESM.zip › 11263_2022_1743_MOESM1_ESM/cat-lion.gif]

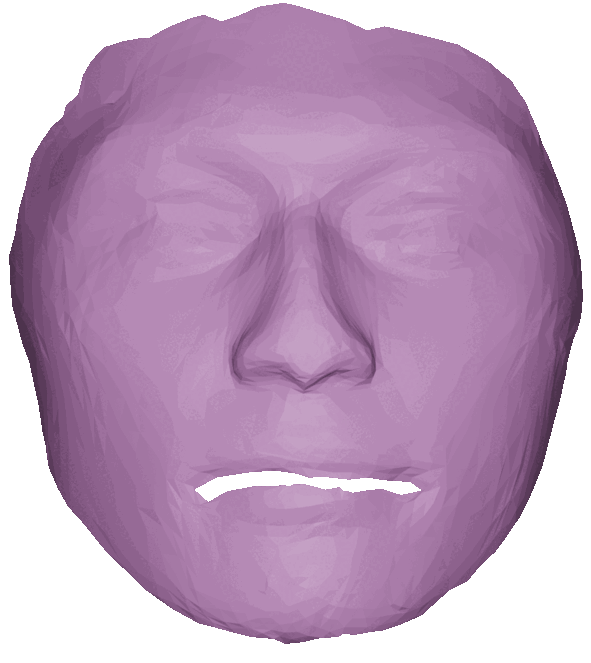

Supplement: Supplementary file 1 — (zip 13912 KB) [file 11263_2022_1743_MOESM1_ESM.zip › 11263_2022_1743_MOESM1_ESM/faces.gif]

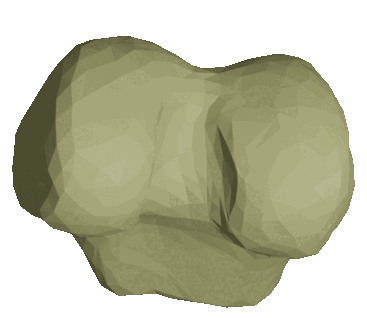

Supplement: Supplementary file 1 — (zip 13912 KB) [file 11263_2022_1743_MOESM1_ESM.zip › 11263_2022_1743_MOESM1_ESM/femur.gif]

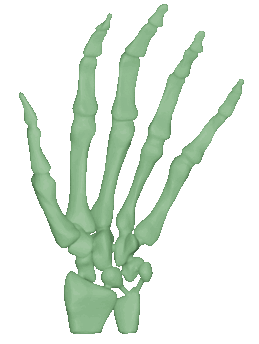

Supplement: Supplementary file 1 — (zip 13912 KB) [file 11263_2022_1743_MOESM1_ESM.zip › 11263_2022_1743_MOESM1_ESM/hands.gif]

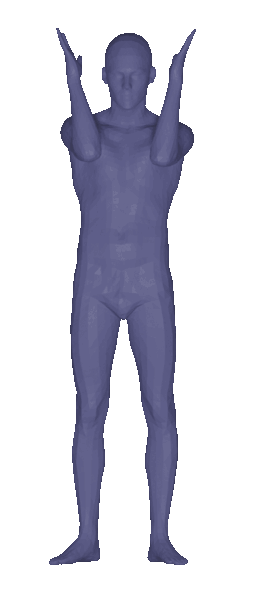

Supplement: Supplementary file 1 — (zip 13912 KB) [file 11263_2022_1743_MOESM1_ESM.zip › 11263_2022_1743_MOESM1_ESM/pose_and_body.gif]

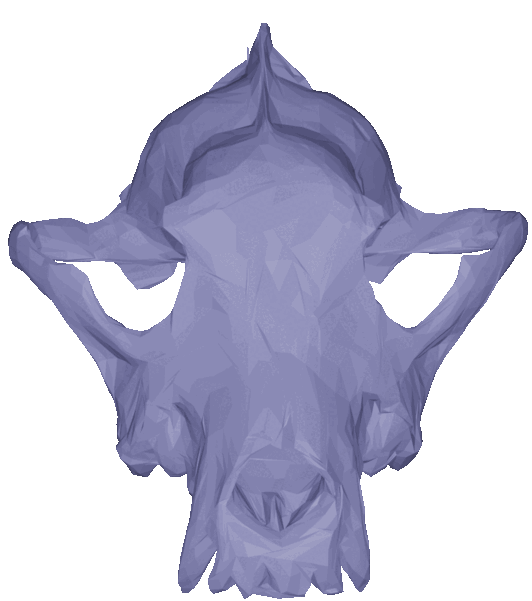

Supplement: Supplementary file 1 — (zip 13912 KB) [file 11263_2022_1743_MOESM1_ESM.zip › 11263_2022_1743_MOESM1_ESM/skulls.gif]
